# African-American esophageal squamous cell carcinoma expression profile reveals dysregulation of stress response and detox networks

**DOI:** 10.1186/s12885-017-3423-1

**Published:** 2017-06-19

**Authors:** Hayriye Verda Erkizan, Kory Johnson, Svetlana Ghimbovschi, Deepa Karkera, Gregory Trachiotis, Houtan Adib, Eric P. Hoffman, Robert G. Wadleigh

**Affiliations:** 1 Institute for Clinical Research, Department of Veteran Affairs Medical Center (VAMC), Washington, D.C., USA; 2 Bioinformatics Neuroscience Group, Information Technology Program, National Institute of Neurological Disorders & Stroke, Bethesda, MD USA; 3 Research Center for Genetic Medicine, Children’s National Medical Center, Washington, D.C. USA; 4 Cardiothoracic Surgery, VAMC, Washington, D.C. USA; 5 Radiology Service, VAMC, Washington, D.C. USA; 6 Present address: School of Pharmacy, Binghamton University – SUNY, Binghamton, NY USA; 7 Oncology Section, Washington DC VAMC, 50 Irving St. NW, Washington DC, 20422 USA

**Keywords:** mRNA expression, Microarray, Down-regulated genes, Up-regulated genes, Pathway analysis, Targeted therapy

## Abstract

**Background:**

Esophageal carcinoma is the third most common gastrointestinal malignancy worldwide and is largely unresponsive to therapy. African-Americans have an increased risk for esophageal squamous cell carcinoma (ESCC), the subtype that shows marked variation in geographic frequency. The molecular architecture of African-American ESCC is still poorly understood. It is unclear why African-American ESCC is more aggressive and the survival rate in these patients is worse than those of other ethnic groups.

**Methods:**

To begin to define genetic alterations that occur in African-American ESCC we conducted microarray expression profiling in pairs of esophageal squamous cell tumors and matched control tissues.

**Results:**

We found significant dysregulation of genes encoding drug-metabolizing enzymes and stress response components of the NRF2- mediated oxidative damage pathway, potentially representing key genes in African-American esophageal squamous carcinogenesis. Loss of activity of drug metabolizing enzymes would confer increased sensitivity of esophageal cells to xenobiotics, such as alcohol and tobacco smoke, and may account for the high incidence and aggressiveness of ESCC in this ethnic group. To determine whether certain genes are uniquely altered in African-American ESCC we performed a meta-analysis of ESCC expression profiles in our African-American samples and those of several Asian samples. Down-regulation of TP53 pathway components represented the most common feature in ESCC of all ethnic groups. Importantly, this analysis revealed a potential distinctive molecular underpinning of African-American ESCC, that is, a widespread and prominent involvement of the NRF2 pathway.

**Conclusion:**

Taken together, these findings highlight the remarkable interplay of genetic and environmental factors in the pathogenesis of African-American ESCC.

**Electronic supplementary material:**

The online version of this article (doi:10.1186/s12885-017-3423-1) contains supplementary material, which is available to authorized users.

## Background

Esophageal cancer is the third leading gastrointestinal malignancy worldwide with greater incidence in males than in females. Patients with esophageal cancer (EC) show limited response to multimodal treatments with an overall five-year survival rate of only about 20% [1]. Due to lack of effective screening for early detection, EC is usually diagnosed at an advanced stage or when metastasis has already occurred. Consistently reliable molecular markers to monitor outcomes remain to be developed [2].

Esophageal cancer has two main histologic subtypes and they arise in two distinct areas of the esophagus. Adenocarcinoma of the esophagus (EAC) is mostly seen in Western countries [3] while esophageal squamous cell carcinoma (ESCC) is predominant in Eastern countries and the eastern part of Africa [3]. Geographical and genomic differences play a significant role in ESCC [4]. In African-Americans, ESCC is the predominant subtype, and the survival rate is worse than in patients of other ethnic groups [5].

The combined action of genetic and environmental factors is believed to underlie the etiology of esophageal cancer. Recent genome-wide association studies, gene expression profiling, DNA methylation and proteomic studies conducted in Japanese and Chinese ESCCs (reviewed in [6]) have identified multiple risk variants and gene signatures associated with ESCC. These studies presented additional evidence for the effect of environmental exposures such as alcohol intake, smoking, opium abuse, hot food and beverage consumption, and diet as risk factors for ESCC [3, 7–11].

Genetic and transcriptome analyses on African-American ESCC have been particularly limited which highlights the lack of understanding of the genetic architecture of ESCC in this ethnic group. In an earlier study of black male ESCC samples, we detected loss of heterozygosity that spanned a significant portion of chromosome 18 [12]. To explore the entire anatomy of the neoplastic genome in black ESCC, we performed comparative genomic hybridization (CGH) on a panel of 17 matched pairs of tumor and control esophageal tissues [13]. Multiple chromosomal gains, amplifications and losses that represent regions potentially involved in etiology defined the pattern of abnormalities in the tumor genome [13]. We noted genomic imbalances that were represented disproportionately in African-American ESCC compared to those reported in ESCC of other ethnic groups including Japanese [14–18], South African black and mixed-race individuals [19], Taiwan Chinese [20], Hong Kong Chinese [21], Chinese in Henan province [22], and Swedes [23].

The preponderance of chromosomal aberrations in African-American ESCC predicts concomitant changes in gene activity during carcinogenesis. We sought to identify dysregulated genes and pathways that could define the expression signature in African-American ESCC by conducting microarray expression profiling in paired squamous esophageal tumors and normal tissue specimens. Here, we report significant differential expression of a wide array of genes involved in multiple pathways that may be crucial to causation and/or progression. Particularly noteworthy is the dysregulation of NRF2 mediated oxidative stress genes and genes that encode drug-metabolizing enzymes and xenobiotics that may, in part, contribute to the aggressive nature of ESCC among blacks.

## Methods

### Samples

Seven paired specimens of the esophagus (tumor and matching non-tumor tissues), each pair derived from the same patient, were collected endoscopically or surgically at the time of diagnosis, frozen and stored at -80 °C until use. Staging indicated that all tumors included in this study were at Stage IV. This study was done under a protocol approved by the Washington D.C. VAMC Institutional Review Board and a written informed consent was obtained from each patient prior to biopsy or surgery. The demographics and risk factors of the patients are listed in the Additional file 1.

### RNA extraction

Tissue samples were subjected to total RNA extraction using TRIzol-reagent (Invitrogen, Carlsbad, CA) and purified with RNeasy Mini kit (Qiagen), according to the manufacturer’s guidelines. The concentration of each RNA sample was determined by NanoDrop spectrophotometer ND-1000 (NanoDrop Technologies, Wilmington, DE). RNA quality was assessed using the Agilent 2100 Bioanalyzer (Agilent Technologies Inc., Santa Clara, CA).

### cRNA preparation and expression profiling

An aliquot of 5 μg of high-quality total RNA from each sample was used to synthesize cDNA and biotinylated cRNA utilizing the Affymetrix GeneChip® Expression 3’Amplification One-Cycle Target Labeling and Control Reagent kit according to manufacturer’s instructions. Biotinylated cRNA was hybridized to Affymetrix Gene-Chips HG U133 Plus 2 (Affymetrix, Santa Clara, CA), washed, stained on the Affymetrix Fluidics station 400 and scanned with a Hewlett Packard G2500A Gene Array Scanner following Affymetrix instructions. All arrays used in the study passed the quality control set by Tumor Analysis Best Practices Working Group [24].

#### Microarray data analysis

The Affymetrix scanner 3000 was used in conjunction with Affymetrix GeneChip Operation Software to generate one. CEL file per hybridized cRNA. These files have been deposited in NCBI Gene Expression Omnibus (GEO) (www.ncbi.nlm.nih.gov/geo/) under the GEO accession number of GSE77861 and are freely available for download.

The Affymetrix Expression Console was used to summarize the data contained across all .CEL files and generate 54,675 RMA normalized gene fragment expression values per file. Quality of the resulting values was challenged and assured via Tukey box plot, covariance-based PCA scatter plot, and correlation-based heat map using functions supported in “R” (www.cran.r-project.org). Lowess modeling of the data (CV ~ mean expression) was performed to characterize noise for the system and define the low-end expression value at which the linear relationship between CV and mean was grossly lost (expression value = 8). Gene fragments not having at least one sample with an expression value greater than this low-end value were discarded as noise-biased. For gene fragments not discarded, differential expression was tested between Tumor and Non-tumor biopsies via paired t-test under Benjamini*–*Hochberg multiple comparison correction condition (alpha = 0.05). Gene fragments having a corrected *P* < 0.05 by this test and an absolute difference of means > = 1.5X were subset as those having differential expression between Tumor and Non-Tumor. Gene annotations for these subset fragments were obtained from IPA (www.ingenuity.com) along with the corresponding enriched functions, enriched pathways, and significant predicted upstream regulators. The analysis pipeline is summarized in the Additional file 2.

### Validation of results by real-time PCR

RT-PCR was performed for *KRT17, PRDCSH, TNFRSF6B, SELK, RAB5B, ALD, RAF* genes. The delta-delta Ct calculation method was used for the quantification of the RT-PCR results.

### Pathway analysis

Ingenuity Pathway Analysis (IPA) (Qiagen- Build version 364,062 M, Content version 26,127,183) was used to determine perturbed pathways. In addition, we performed IPA to identify perturbed pathways affected in ESCC from different ethnic groups by utilizing publicly available datasets of ESCC mRNA expression microarrays including GSE17351 [25], GSE20347 [26], GSE23400 [27], GSE29001 [28], GSE33426 [28], GSE33810 [29] and GSE45670 [30] from the GEO repository (http://www.ncbi.nlm.nih.gov/geo/). The characteristics of these studies such as sample size, tissue storage, and control tissue type are presented in the Additional file 3. The differentially expressed gene lists were obtained by the analysis with GEO2R (http://www.ncbi.nlm.nih.gov/geo/geo2r/). The *p*-values were adjusted with Benjamini and Hochberg correction.

## Results

**Fig. 1:**
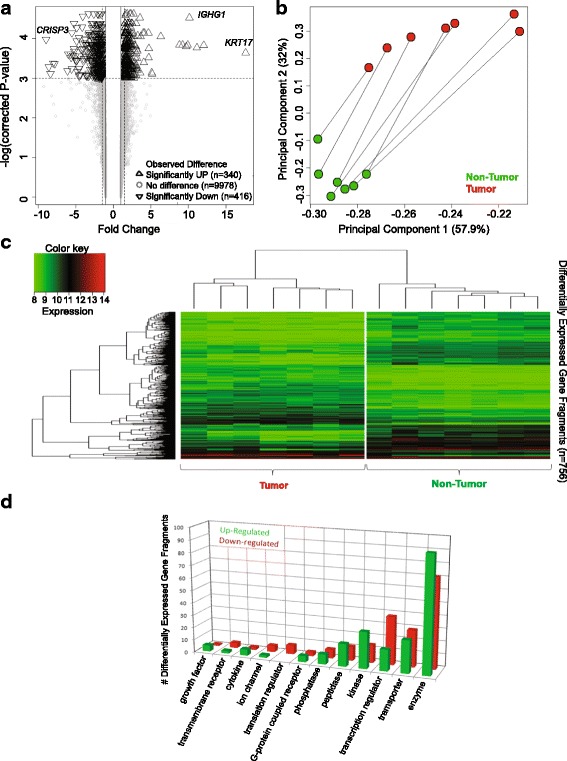
Gene expression differences observed between paired Esophageal Tumor and Non-Tumor biopsies for seven patients. **a** Volcano Plot depicting the differential expression testing results for 10,734 gene fragments. Gene fragments having significant difference in expression between Tumor and Non-Tumor where the magnitude of difference is also > = 1.5X are represented as triangles (*n* = 756). **b** Covariance-based Principal Component Analysis (PCA) scatter plot depicting the paired sample relationships when the 756 gene fragments identified to have significant difference in expression between Tumor and Non-Tumor are used. **c** Correlation-based clustered heat map depicting the sample relationships (x-axis) when the 756 gene fragments identified to have significant difference in expression between Tumor and Non-Tumor (y-axis) are used. **d** *Bar plot* describing the breakdown of the 756 gene fragments identified to have significant difference in expression between Tumor and Non-Tumor by protein type (where known).

Transcriptome profiling of African-American ESCC tumors versus adjacent normal esophageal tissues revealed significant differential expression of 756 genes comprising 340 over-expressed and 416 under-expressed loci that were detected by 460 and 558 gene probes, respectively (Additional file 4). A volcano plot displayed genes that underwent the highest alteration in expression (Fig. 1a). Among the most strongly up-regulated genes are keratin 17 (*KRT17*), immunoglobulin genes including *IGHG1* and ornithine decarboxylase 1 (*ODC1*). Genes that showed a huge loss of expression included cysteine-rich secretory protein 3 (*CRSP3*) and sciellin (*SCEL*). Experimental validation of microarray results through a real-time PCR assay on RNA derived from the same original samples for selected up-regulated (*KRT17, PRDCSH, TNFRSF6B*) and down-regulated (*SELK, RAB5B, ALD, RAF*) genes supported the microarray data (data not shown).

Principal component analysis of differentially expressed genes indicated the magnitude of the co-variance between paired tumor and non-tumor samples of each patient (Fig. 1b). The first principal component contributed 57.9% of the variance among the samples. Correlation-based clustering of all differentially expressed genes distinguished clearly tumor from the corresponding non-tumor tissues (Fig. 1c).

### Perturbed pathways and networks in African-American ESCC

To determine the overall biological impact of the widespread transcriptional aberration in African-American ESCC, we performed pathway and network analysis on significantly dysregulated using Ingenuity Pathway Analysis (IPA). The majority of differentially expressed genes encoded a diversity of enzymes (Fig. 1d). Genes that coded for transporters, transcription regulators, phosphatases, translation regulators, ion channels and transmembrane receptors were among those that were most prominently down-regulated (Fig. 1d).

**Fig. 2:**
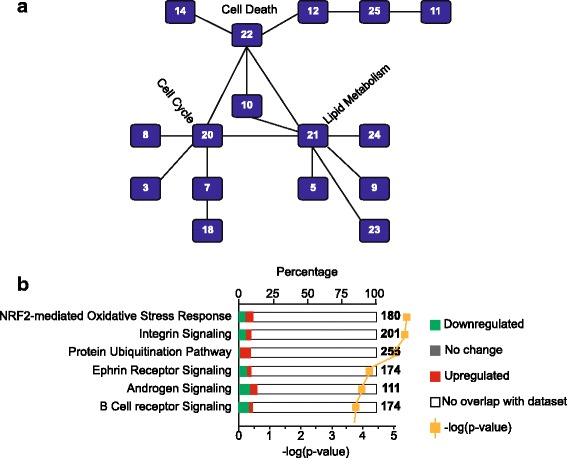
Ingenuity Pathway Analysis (IPA) of ESCC.** a** Interconnected canonical pathways. Pathway 20 (injury and abnormalities and cell cycle), pathway 21 (carbohydrate and lipid metabolism, and molecular transport), and pathway 22 (cell death and survival pathways) serve as hub for interconnected canonical pathways. **b** The enriched canonical pathways in ESCC by IPA. The most enriched pathways represented the higher –log(*p*-value). The *white bar* represents the genes that do not overlap with the data set. *Green bar* represents genes that are down-regulated and red bar represents genes that are up-regulated. The *gray bar* demonstrates the genes without any change in expression.

IPA detected the enrichment of 25 networks (Fig. 2, Additional file 5), 14 of which were interconnected. Networks 20, 21, and 22 displayed linkage to at least five other networks representing the highest number of interconnections. The cell cycle and organismal injury and abnormalities were the constituent pathways of network 20. Network 21 included carbohydrate and lipid metabolism and molecular transport, and network 22 comprised cell death and survival pathways. (The complete list of genes in these networks is presented in Additional file 5).

Fifteen canonical pathways were significantly enriched in African-American ESCC and the top three included NRF2-mediated oxidative stress pathway, integrin signaling and protein ubiquitination, in that order (Fig. 2b, Additional file 6). The gene constituents of these pathways are presented in Additional file 7. These results suggest that African-American ESCC is underpinned by a dysregulation of genes that play an important role in oxidative stress and xenobiotic metabolic responses.

### Activation of NRF2 perturbs stress response and detoxification pathways in ESCC

Enriched pathways involving stress response, xenobiotic metabolism, and toxic response are noteworthy because smoking and alcohol consumption have been consistently shown to be strong contributing factors in ESCC etiology. It was therefore important to focus on pathways involved in detox networks.

**Fig. 3:**
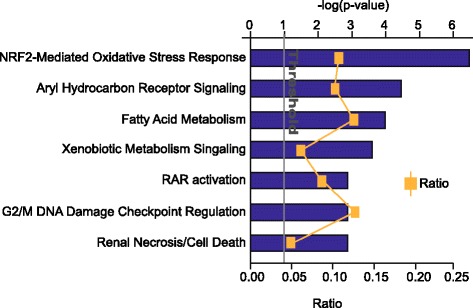
The toxicology chart summarizes the enrichment of detoxification pathways enriched in our dataset by IPA. Ingenuity Pathway Analysis (IPA) identified NRF2-mediated oxidative stress response pathway as the most enriched toxicology pathway**.** *Blue bar* represent –log(*p*-value) and the ratio is the number of genes characterized in the dataset compared to the total number of genes belonging to that pathway

The NRF2-mediated oxidative stress response pathway showed the highest enrichment (with a –log(p) of 6.25), in general, and in the toxicology panel as well (Fig. 3). NRF2 pathway is one of the primary mediators of detoxification and metabolism responses. Transcriptional targets of NRF2 include genes involved in alcohol metabolism such as *ADH7, AKR1B1, ALDH3A1,* and *ALDH7A1,* all of which are differentially expressed in our dataset (Additional file 8). Other targets that showed altered expression in African-American ESCC include genes with a wide range of function: *MGST2, ABCC1, ABCC5, GCLC GPX4, ACOX1, BLVRA, FTL1, CEBPB, ACLY, ELOVL5, FABP5, ACAA1B*.

IPA predicted that 19 upstream regulators are activated in our dataset (Table 1 and Additional file 9). Nuclear factor-erythroid 2 p45-related factor 2 gene, *NFE2L2,* a known upstream regulator of the NRF2 pathway was predicted to have the highest activation z-score of 3.796, followed by MEK, LDL, and CTNNB1 pathways, with decreasing z-scores. In addition, *MYC* was predicted to be an activated upstream regulator (Additional file 9).

**Table 1:** Comparison of the predicted upstream regulatory pathways in ESCC

GSE77861			GSE17351			GSE33810			GSE20347		
Gene	z-score	-log(p)	Gene	z-score	-log(p)	Gene	z-score	-log(p)	Gene	z-score	-log(p)
NFE2L2	3.8	7.5	**NUPR1**	4.7	10.1	mir-8	2.6	1.2	**RABL6**	5.8	26.7
MEK	2.7	1.2	**CLDN7**	3.9	7.9	**let-7**	2.6	0.3	**FOXM1**	4.4	14.2
LDL	2.8	0.5	**MYOCD**	3.8	6.0	LYN	2.4	0.6	IgG complex	3.7	21.9
**CTNNB1**	2.6	1.6	**NR3C1**	3.7	4.2	**IL10RA**	2.3	1.0	MITF	3.5	15.8
**RABL6**	2.5	2.1	I**RGM1**	3.5	7.0	**TRIM24**	2.4	0.1	FOXO1	3.4	11.3
**FOXM1**	2.4	1.2	**BNIP3L**	3.4	8.2	miR-1-3p	2.2	0.8	RARA	3.3	16.2
CBX5	2.3	2.4	**RBL2**	3.2	4.8	IFI16	2.2	0.4	TLR7	3.3	4.2
ANGPT2	2.3	2.3	**TP53**	3.2	21.1	ZBTB16	2.2	2.0	PRL	3.3	8.1
PLIN5	2.2	2.2	**IL1RN**	3.1	4.3	miR-10	2.2	0.4	**IFNL1**	3.2	10.0
ANXA7	2.2	2.4	**SRF**	3.0	5.7	**CDKN2A**	2.1	0.1	**TGFB1**	3.2	46.3
**TP53**	−3.1	18.4	**CSF2**	−5.6	9.3	**ERBB2**	−3.2	8.2	**TP53**	−5.3	38.7
IL13	−3.0	0.7	**RABL6**	−4.1	8.9	SHH	−3.1	7.8	**NUPR1**	−4.4	19.5
**CDKN2A**	−3.0	2.6	SPP1	−4.1	5.3	IGFBP2	−3.1	7.5	**SPDEF**	−4.0	8.7
**CD28**	−2.8	2.0	**EGFR**	−3.8	14.0	TGFB3	−2.9	7.5	**KDM5B**	−3.4	12.6
EHF	−2.5	2.3	ERK1/2	−3.8	9.2	ERG	−2.9	7.0	HSF1	−3.3	6.7
**CLDN7**	−2.4	3.9	**EGF**	−3.7	9.9	**CCTNB1**	−2.7	7.1	CDKN1A	−3.3	9.5
**let-7**	−2.2	0.5	**HGF**	−3.6	17.1	CREB	−2.6	1.7	**CLDN7**	−3.1	6.7
ESRRA	−2.2	1.4	**TNF**	−3.4	3.9	WNT1	−2.5	0.6	BTK	−2.9	5.0
TCF3	−2.0	0	**E2F1**	−3.4	7.9	**CCND1**	−2.5	2.9	WISP2	−2.9	8.6
FGFR1	−2.0	0.8	**FN1**	−3.4	4.7	ERG2	−2.5	0.9	E2F6	−2.8	4.3
GSE29001			GSE33426			GSE45670			GSE23400		
Gene	z-score	-log(p)	Gene	z-score	-log(p)	Gene	z-score	-log(p)	Gene	z-score	-log(p)
**RABL6**	5.8	25.2	**TGFB1**	7.7	42.9	**TNF**	5.3	24.0	**CSF2**	5.0	14.4
HGF	5.7	39.5	**TNF**	7.2	15.1	**ERBB2**	4.9	21.3	**RABL6**	4.7	19.7
**VEGF**	5.3	32.7	**VEGF**	7.0	15.0	CSF	4.2	4.8	**VEGF**	4.4	32.3
**FOXM1**	5.0	19.4	**HGF**	6.9	22.0	**EGFR**	3.9	13.6	**HGF**	4.2	38.8
**CSF2**	5.0	30.7	**ESR1**	6.9	42.0	**IFNL1**	3.8	5.8	**FOXM1**	4.0	16.0
**E2F1**	4.6	28.1	**EGF**	6.5	12.0	I**FNG**	3.7	21.3	**ESR1**	3.9	31.1
**TBX2**	4.4	13.0	**CSF2**	6.5	14.0	**CCND1**	3.7	18.5	**ERBB2**	3.7	56.4
E2F group	4.2	15.8	**CTNNB1**	6.3	13.0	IL1A	3.6	15.5	**FN1**	3.7	7.5
IFNA1	3.7	6.4	SMARCA4	6.2	11.0	**RABL6**	3.6	5.1	**TBX2**	3.6	12.2
**IFNL1**	3.6	13.0	**IFNG**	5.9	15.0	OSM	3.5	15.6	**EGF**	3.4	32.6
**NUPR1**	−6.2	15.6	**let-7**	−6.0	19.0	GATA4	−4.8	11.6	**TP53**	−5.2	53.0
**let-7**	−5.0	17.0	CD3	−5.1	15.0	**IL10RA**	−4.3	7.4	**CDKN2A**	−4.3	10.5
**KDM5B**	−4.5	10.7	**SPDEF**	−4.2	8.0	**MYOCD**	−3.9	11.2	**let-7**	−3.9	16.0
**IRGM**	−4.5	14.8	**KDM5B**	−4.1	7.5	**IL1RN**	−3.6	8.1	**RB1**	−3.8	17.7
**TP53**	−4.2	54.2	**IRGM1**	−4.0	9.0	**IRGM1**	−3.5	6.5	**NR3C1**	−3.5	7.9
**SPDEF**	−4.1	10.2	**TRIM24**	−4.9	4.0	HAND2	−3.3	5.7	**SPDEF**	−3.4	8.5
**RBL2**	−4.0	13.7	**RB1**	−3.9	14.0	**SRF**	−3.3	11.1	PPARG	−3.3	13.0
**BNIP3L**	−4.0	17.3	**RBL2**	−3.9	7.2	ACKR2	−3.2	5.3	let-7a-5p	−3.3	4.3
**CDKN2A**	−3.9	7.3	**IL1RN**	−3.8	2.2	PTEN	−3.2	5.0	**IRGM1**	−3.2	7.2
**TRIM24**	−3.8	18.2	**CD28**	−3.6	8.2	POU5F1	−3.0	2.4	CR1L	−3.1	8.9

The upstream regulatory pathways represented more than one study in the meta-analysis indicated in bold

The TP53 regulatory pathway was predicted to be the most inhibited with a z-score of −3.113 and a *p*-value of 4.05E-19 (Table 1). In our sample, 99 differentially expressed genes were downstream of the TP53 pathway (Additional file 10). Inhibition of the TP53 pathway is a hallmark of carcinogenesis and is predicted in our ESCC dataset, as well.

### Functional meta-analysis of gene expression of ESCC in diverse ethnic groups

To determine whether African-American ESCC implicates genes that are unique or shared by ESCC of other ethnic groups, we performed a meta-analysis that included our African-American ESCC expression data and data from seven studies published in publicly available datasets in the GEO database. We note that our expression profiling data is the first such study in African-American ESCC to be deposited in the GEO repository. ESCC expression profiles in GEO included those generated in Japan (GSE17351) [25], Hong Kong, China (GSE33810) [29] and from various parts of China (GSE23400 [27], GSE20347 [26], GSE45670 [30], GSE33426 [28], and GSE29001 [28]). Ten genes that underwent the highest changes in expression in these studies are listed in the Additional file 11. Of the up-regulated genes, *KRT17* was over-expressed in two other studies, the rest of the up-regulated genes were ornithine decarboxylase 1 (*ODC1*), Profilin 2 (*PFN2*), Glycoprotein Nmb (*GPNMB*). Six out of 10 down-regulated genes (*CRISP3, TMPRSS11B, CLCA4, SCEL, SLURP1, KRT78*) were shared with four other studies.

**Fig. 4:**
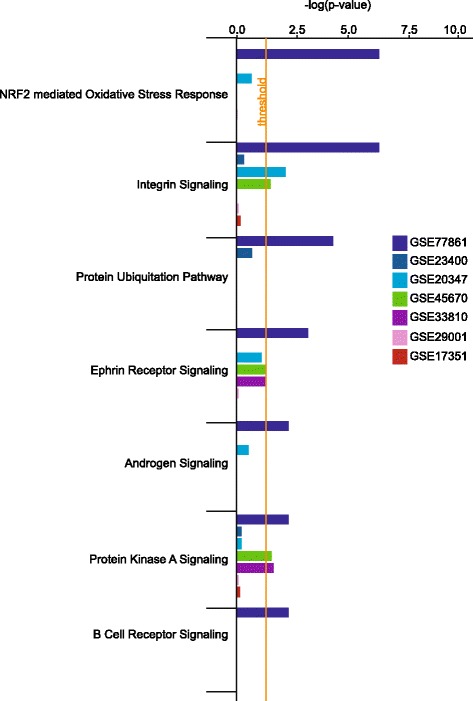
Meta-analysis of the most enriched pathways in ESCC. *Dark navy bars* represent our dataset. *Dark blue, blue, green, purple, pink*, and *red* bars represent the data sets of GSE23400, GSE20347, GSE45670, GSE33810, GSE29001, GSE17351, respectively.

Analysis of the functional outcome of expression profiles from all microarray studies showed that NRF2-mediated oxidative stress pathway was significantly enriched only in our dataset (Fig. 4). Likewise, the significant enrichment of ubiquitination, androgen, and B- cell receptor signaling pathways was revealed only in our dataset. Integrin, ephrin receptor and protein kinase A signaling pathways were shared by at least two or more studies at or above the significance threshold.

**Fig. 5:**
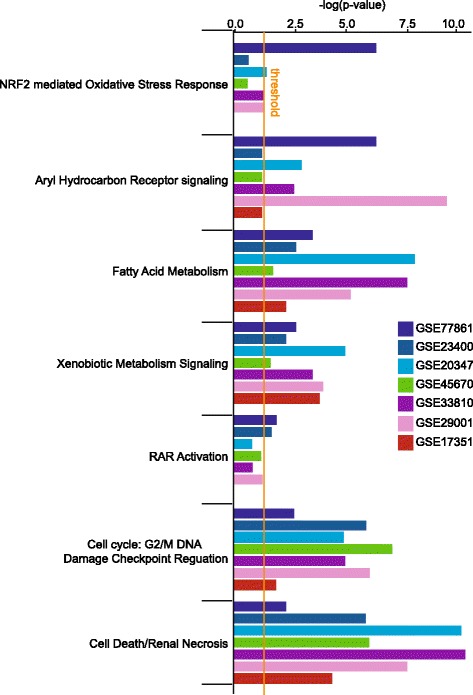
Comparison of the toxicology pathway indicated the enrichment of NRF2 pathway in our dataset. Dark navy bars represent our dataset. *Dark blue, blue, green, purple, pink*, and *red* bars represent the data sets of GSE23400, GSE20347, GSE45670, GSE33810, GSE29001, GSE17351, respectively.

It was important to examine the dysregulation of genetic components of the detox networks in the ESCC microarray expression datasets. All studies showed enrichment of toxicology pathways than other signaling pathways (Fig. 5). Interestingly, our dataset contained the highest number of genes in the NRF2-mediated oxidative stress response pathway while in other studies this number was either at or below the significance threshold. Aryl hydrocarbon receptor, fatty acid metabolism, xenobiotic metabolism signaling, G2/M DNA damage checkpoint regulation and cell death genes were significantly perturbed in all studies. In our dataset (GSE77861) and in GSE23400 [27], the number of genes in retinoic acid receptor signaling was above the significance threshold.

### Meta-analysis of the upstream regulatory pathways of ESCC in various ethnic groups

Meta-analysis of all available ESCC gene expression profile datasets showed a distinctive upstream regulatory pathway in African-Americans that highlighted a significant enrichment of the NRF2 mediated oxidative stress response pathway (Table 1). The activated pathways such as CBX5, insulin, MEK, NFE2L2, ANXA7, HSF2, NFE2L1, and PLIN5 were either uniquely represented in our study or shared with only one other study. Six out of eight datasets predicted the activation of upstream pathways of E2F and RABL6 although the rankings of z-score of these pathways were diverse (Table 1 and Additional file 9). FOXM1 was also projected as one of the common activated upstream pathways. Regardless of the z-score rankings, the activation of angiopoietin 2 pathway is the third highly represented upstream pathway in five of the studies (Additional file 9). The activation of fibronectin, and beta-catenin pathways as upstream regulators was revealed in five studies that included ours.

The predicted inhibited upstream pathways were divergent among the studies. While the TP53 pathway was predicted to be the top inhibited pathway in our study, the most common inhibited pathways including CDKN1A, IRF4, KDM5B, ACKR2, BNIP3L, DYRK1A were found in all datasets except in our study. In contrast, our dataset exclusively demonstrated the inhibition of FGFR1, ESRRA, EHF, and IL13 pathways.﻿ 

## Discussion

ESCC is the predominant esophageal carcinoma subtype worldwide occurring in specific geographic areas and in various countries including China, Japan, Iran, Italy and France [8, 31]. In the United States, a high incidence of ESCC has been reported in the District of Columbia and coastal areas of the southern states [32]. ESCC occurs at a 5-fold greater frequency among African-Americans than among white Americans while the converse has been observed for EAC [7, 33]. Even though five-year survival rates increased in both whites and black between 2004 and 2010, the mortality rate for esophageal carcinoma is still far greater in blacks than among whites [33–35]. Notably, in recent years, an increased incidence of EAC has been observed, particularly among whites [1, 34]. Altogether, these distinctive features indicate geographic and racial disparities in esophageal cancer [31].

We conducted a transcriptome analysis to identify the molecular repertoire involved in esophageal squamous cell carcinoma in African-American males. To our knowledge, this study is the first to investigate and analyze the global gene expression pattern of stage IV ESCC in African-Americans.

Heavy alcohol consumption, cigarette smoking, and poor diet are environmental risk factors for ESCC. Our findings in African-American ESCC reveal dysregulation of genes involved in detox networks, including NRF2 pathway, which is a primary mediator of detoxification and metabolism responses (Additional file 5) [36]. Nuclear factor-erythroid 2 p45-related factor 2 (*NFE2L2*) gene encodes a transcription factor NRF2 that regulates the transcription of antioxidant/electrophile response element (ARE)-containing target genes in response to oxidative and/or toxic environmental changes. The NRF2 pathway also regulates wound healing, resolution of inflammation, autophagy, ER stress response and unfolded protein response [37], apoptosis, differentiation of keratinocytes [38] and the embryonic development of the esophagus in response to growth factor-induced ROS production [39, 40].

The role of NRF2 pathway is cancer-type dependent. NRF2 protects against chemical carcinogen-induced carcinogenesis in the stomach, bladder and skin [41]. However, NRF2 activation plays an oncogenic role in lung, head and neck, ovarian and endometrial cancers [41]. Previous studies conducted in Asian samples demonstrated that higher expression of NRF2 is positively correlated with lymph node metastasis and drug resistance in ESCC [42]. Mutations in *NFE2L2* confer malignant potential and resistance to therapy in advanced ESCC [43]. However, only 10% of Asian ESCC carry mutations in the *NFE2L2* gene or its negative regulator *KEAP1* [44]. Consistent with this data, our meta-analysis of gene expression profiles only showed a modest involvement of NRF2 in toxicology pathways in Asian ESCC datasets. IPA demonstrated the enrichment of NRF2 pathway in ESCC with high confidence in our dataset, suggesting a unique molecular signature of African-American ESCC. The significance of NRF2 pathway in African-American ESCC merits further functional evaluation.

In our CGH data, we previously found a loss of 7q in >50% of ESCC from African-American males [13]. Transcriptome mapping identified four genes located in the 7q21.1–22.3 region among which is the cytochrome P450 gene cluster that includes *CYP3A5, CYP3A7*, *CYP3A4,* and *CYP3A43.* It is noteworthy that our analysis indicates a significant loss of expression of *CYP3A5* in addition to the down-regulation of three other genes that encode cytochrome P450 enzymes. It is well established that CYP3A enzymes metabolize more than half of the drugs used clinically [45]. Cytochrome P450 enzymes are also active in metabolizing toxic compounds thus their loss potentially contributes to carcinogenesis.

The persistent metabolic imbalance and tumor promoters found in cigarette smoking activate growth-promoting, cancerous conditions. Thus, the continual activation of NRF2 pathway could provide an adaptation mechanism to environmental toxicant especially in cancers [37]. Aryl hydrocarbon signaling, fatty acid, and xenobiotic metabolism also share some of the proteins that function in the NRF2 pathway. Therefore, the effect of the dysregulated NRF2 pathway may amplify the impairment of the dynamics of these pathways. In addition to response to toxins, NRF2 might promote cell proliferation of cancer cell by reprogramming metabolism to anabolic pathways [46]. However, further studies are required to investigate the causal association of NRF2 pathway in the esophageal tumor development in African-Americans. Future genomic studies are important to evaluate the mutational spectra of *NFE2L2* or *KEAP1* in African-American ESCC.

Recent studies that outlined the genomic and molecular characterization of esophageal carcinoma in the Asian population suggested the dysregulation of the receptor tyrosine kinase (RTK)-MAPK-PI3K, NOTCH, Hippo, cell cycle, and epigenetic pathways as the primary molecular mechanism of ESCC [44, 47]. The amplification or over-expression of *FGFR1, MET*, *EGFR, ERBB2, ERBB4,* and *IL7R* was observed in the majority of the patients and has been suggested as main drivers for the ESCC tumorigenesis [47]. Our meta-analysis of ESCC expression datasets indicated that the activation of growth factors and or their receptors, RABL6, FOXM1, CCND1, and CTNNB1 are upstream signaling drivers of the cellular growth of ESCC.

The upstream regulatory role of RABL6 was predicted in six out of eight ESCC datasets. *RABL6* gene encodes a member of the Ras superfamily of small GTPases. The encoded protein RABL6, also known as RBEL or PARF, binds to both GTP and GDP and may play a role in cell growth and survival. Overexpression of this gene may play a role in breast, and pancreatic cancer tumorigenesis [48–50]. Functional analysis of RABL6 in ESCC warrants further study.

The most common inhibited upstream regulatory pathways are TP53 and KDM5B across most of the ESCC datasets. Studies have shown that TP53 negatively regulates NRF2-mediated gene expression [51]. The down-regulation of TP53 could synergistically sustain the activation of NRF2 seen in African-American ESCC. We previously identified a single nucleotide mutation of *SCEL* gene in both normal and squamous cell carcinoma of esophagus in African-Americans [52]. In our present study, *SCEL* is significantly under-expressed in African-American ESCC, and thus could play a role in squamous cell carcinogenesis as suggested by the down-regulation of this gene in larynx and hypopharynx [53], and in tongue squamous cell carcinoma [54].

The diversity among the inhibited upstream pathways implies the variety of susceptibility loci remain to be discovered in ESCC tumorigenesis, particularly the contribution of the deregulation of immune components. Given the differences in enriched pathways displayed by ESCC in various ethnic groups, it is possible that different genetic backgrounds have dissimilar responses to various environmental exposures. [55, 56].

Conceivably, our findings unmasked only a restricted view of the processes that are compromised in ESCC given the inherent limitations of microarray-based transcriptome profiling, the small sample size that was analyzed and incomplete modeling of biological reactions due to lack of functional data. However, the present study uncovered salient mechanistic aspects of the squamous esophageal cellular system in African-Americans, which to our knowledge, have not been described previously.

## Conclusion

Our expression profiling study and pathway analysis suggest a widespread and prominent disruption of detox networks as revealed by the involvement of the NRF2 pathway and loss of detoxifying enzymes as a potential distinctive molecular mechanism in African-American esophageal squamous cell carcinogenesis.

## Additional files

Additional file 1: The list of the demographics and risk factors of the patients. (XLSX 9 kb)

Additional file 2: Analysis pipeline. (EPS 830 kb)

Additional file 3: The characteristics of studies included in Meta-analysis. (XLSX 11 kb)

Additional file 4: The gene expression data file. (XLSX 118 kb)

Additional file 5: The description of 25 networks found enriched in our data set by IPA. (XLSX 13 kb)

Additional file 6: Fifteen canonical pathways that were significantly enriched in African-American ESCC. (EPS 1053 kb)

Additional file 7: The list of the gene constituents of fifteen canonical pathways that were presented in Additional file 5. (XLSX 43 kb)

Additional file 8: The role of NRF2 pathway and transcriptional targets of NRF2 which are differentially expressed in our dataset. (EPS 994 kb)

Additional file 9: The meta-analysis of ESCC gene expression studies. (XLSX 438 kb)

Additional file 10: The schematic representation of 99 differentially expressed genes that were downstream of the TP53 pathway. (EPS 27906 kb)

Additional file 11: The table of top ten of the most differentially expressed genes in the studies contributed in the meta-analysis. (EPS 1532 kb)

## Abbreviations

ABCC1: ATP binding cassette subfamily C member 1
ABCC5: ATP binding cassette subfamily C member 5
ACAA1B: acetyl-Coenzyme A acyltransferase 1B
ACKR2: atypical chemokine receptor 2
ACLY: ATP citrate lyase
ACOX1: acyl-CoA oxidase 1
ADH7: alcohol dehydrogenase 7 (class IV)
AKR1B1: aldo-keto reductase family 1 member B
ALD: ABCD1 ATP binding cassette subfamily D member 1
ALDH3A1: aldehyde dehydrogenase 3 family member A1
ALDH7A1: aldehyde dehydrogenase 7 family member A1
ANXA7: Annexin 7
ARE: antioxidant/electrophile response element
BLVRA: biliverdin reductase A
BNIP3L: BCL2 interacting protein 3 like
CBX5: chromobox 5
CCND1: cyclin D1
CDKN1A: cyclin dependent kinase inhibitor 1A
CEBPB: CCAAT/enhancer binding protein beta
CGH: comparative genomic hybridization
CLCA4: chloride channel accessory 4
CRSP3: cysteine-rich secretory protein 3
CTNNB1: catenin beta 1
CYP3A4: cytochrome P450 family 3 subfamily A member 4
CYP3A43: cytochrome P450 family 3 subfamily A member 43
CYP3A5: cytochrome P450 family 3 subfamily A member 5
CYP3A7: cytochrome P450 family 3 subfamily A member 7
DYRK1A: dual specificity tyrosine phosphorylation regulated kinase 1A
EAC: Adenocarcinoma of esophagus
EC: esophageal cancer
EGFR: Epidermal growth factor receptor
EHF: ETS homologous factor
ELOVL5: ELOVL fatty acid elongase 5
ER: Endoplasmic reticulum
ERBB2: erb-b2 receptor tyrosine kinase 2
ERBB4: erb-b2 receptor tyrosine kinase 4
ESCC: esophageal squamous cell carcinoma
ESRRA: estrogen related receptor alpha
FABP5: fatty acid binding protein 5
FGFR1: fibroblast growth factor receptor 1
FOXM1: forkhead box M1
FTL1: ferritin light polypeptide 1
GCLC: glutamate-cysteine ligase catalytic subunit
GEO: Gene Expression Omnibus
GPX4: glutathione peroxidase 4
HSF2: heat shock transcription factor 2
IGHG1: immunoglobulin
IL13: interleukin 13
IL7R: interleukin 7 receptor
IPA: Ingenuity Pathway Analysis
IRF4: interferon regulatory factor 4
IRGM1: immunity related GTPase M
KDM5B: lysine demethylase 5B
KEAP1: kelch like ECH associated protein 1
KRT17: keratin 17
KRT78: Keratin 78
MAPK: MAP kinase
MEK: MAP kinse-ERK kinase
MET: MET proto-oncogene, receptor tyrosine kinase
MGST2: microsomal glutathione S-transferase 2
NCBI: The National Center for Biotechnology Information
NFE2L1: nuclear factor erythroid-2–related factor 1
NFE2L2: Nuclear factor-erythroid 2 p45-related factor 2
NRF2: nuclear factor erythroid-2–related factor 2
ODC1: ornithine decarboxylase 1
PCA: Principal Component Analysis
PI3K: phosphatidylinositol-4,5-bisphosphate 3-kinase
PLIN5: perilipin 5
RAB5B: RAB5B, member RAS oncogene family
RABL6: RAB, member RAS oncogene family-like 6
RABL6: RAB, member RAS oncogene family-like 6
RAF: Raf-1 proto-oncogene, serine/threonine kinase
RTK: receptor tyrosine kinase
RT-PCR: Real time polymerase chain reaction
SCC: Squamous cell carcinoma
SCEL: sciellin
SELK: selenoprotein K
SLURP1: secreted LY6/PLAUR domain
TMPRSS11B: transmembrane protease, serine 11B
TNFRSF6B: Tumor necrosis factor receptor superfamily member 6b
